# Long-term monitoring of SARS-CoV-2 RNA in wastewater of the Frankfurt metropolitan area in Southern Germany

**DOI:** 10.1038/s41598-021-84914-2

**Published:** 2021-03-08

**Authors:** Shelesh Agrawal, Laura Orschler, Susanne Lackner

**Affiliations:** Chair of Wastewater Engineering, Institute IWAR, Technical University of Darmstadt, Franziska-Braun-Straße 7, 64287 Darmstadt, Germany

**Keywords:** Environmental impact, Public health, Epidemiology

## Abstract

Wastewater-based epidemiology (WBE) is a great approach that enables us to comprehensively monitor the community to determine the scale and dynamics of infections in a city, particularly in metropolitan cities with a high population density. Therefore, we monitored the time course of the SARS-CoV-2 RNA concentration in raw sewage in the Frankfurt metropolitan area, the European financial center. To determine the SARS-CoV-2 RNA concentration in sewage, we continuously collected 24 h composite samples twice a week from two wastewater treatment plant (WWTP) influents (Niederrad and Sindlingen) serving the Frankfurt metropolitan area and performed RT-qPCR analysis targeting three genes (N gene, S gene, and ORF1ab gene). In August, a resurgence in the SARS-CoV-2 RNA load was observed, reaching 3 × 10^13^ copies/day, which represented similar levels compared to April with approx. 2 × 10^14^ copies/day. This corresponds to a continuous increase again in COVID-19 cases in Frankfurt since August, with an average of 28.6 incidences, compared to 28.7 incidences in April. Different temporal dynamics were observed between different sampling points, indicating local dynamics in COVID-19 cases within the Frankfurt metropolitan area. The SARS-CoV-2 RNA load to the WWTP Niederrad ranged from approx. 4 × 10^11^ to 1 × 10^15^ copies/day, the load to the WWTP Sindlingen from approx. 1 × 10^11^ to 2 × 10^14^ copies/day, which resulted in a preceding increase in these loading in July ahead of the weekly averaged incidences. The study shows that WBE has the potential as an early warning system for SARS-CoV-2 infections and a monitoring system to identify global hotspots of COVID-19.

## Introduction

The ongoing pandemic of the coronavirus disease 2019 (COVID-19) is a public health emergency of global concern and is expressed by symptoms like fever, myalgia, fatigue, and dry cough. The disease, caused by severe acute respiratory syndrome coronavirus 2 (SARS-CoV-2), emerged in China in December 2019. The World Health Organization (WHO) declared it a pandemic on March 11th 2020, due to its worldwide spread^1^. The disease has now been reported in over 213 countries, with more than 40 million confirmed cases. The pandemic has caused nationwide lockdowns in many countries and contact restrictions to prevent the spread of the disease^2^.

In the last couple of months, wastewater epidemiology (WBE) has emerged as a promising approach for early warning of disease outbreaks and providing information for the public, especially if patients are asymptotic. Recent studies confirmed the detection of SARS-CoV-2 in feces and urine from positively tested patients^3,4^, which implies that SARS-CoV-2 RNA is present in the influent of wastewater treatment plants (WWTPs)^5^.

The potential advantage of environmental surveillance in WBE is to enable predicting the overall status of a given catchment area with much less effort than clinical surveillance. WBE can provide insight into the outbreak situation in the entire catchment area by testing the wastewater sample over time. In contrast, clinical surveillance requires more time and cost for sample collection and testing. An additional big advantage of WBE is capturing people with asymptomatic and pre-symptomatic infections, who may not be included in clinical surveillance. Several studies have already proved that wastewater monitoring can detect outbreaks of norovirus and poliovirus earlier than clinical surveys^6–9^.

Preliminary studies have reported the detection of SARS-CoV-2 RNA in wastewater in the Netherlands^10^, USA^11^, Australia^12^, and Italy^13^. One of the first studies based on surveillance of COVID-19 in wastewater was performed in Australia, and SARS-CoV-2 RNA was detected in two samples within six days of the same WWTP with both qPCR and sequencing^12^. In the Netherlands, researchers tested sewage of six cities and the airport for SARS-CoV-2 RNA, targeting either the nucleocapsid (N) gene or the envelope (E) gene^10^. The results showed that the sewage samples were tested positive for the N gene in March 2020. In Italy, a research group studied twelve influent sewage samples from the WWTPs in Milan and Rome between February and April, and reported that 6 out of 12 samples were positive^13^.

In this study, the goal was to establish a WBE surveillance system for SARS-CoV-2 in a metropolitan area in Southern Germany (Frankfurt am Main) and to use this data as a warning system in the future. WWTP data can add valuable information and aid decision-making on further public and societal restrictions or easing with increasing or decreasing virus concentration.

## Results

### Epidemiological surveillance

**Figure 1:**
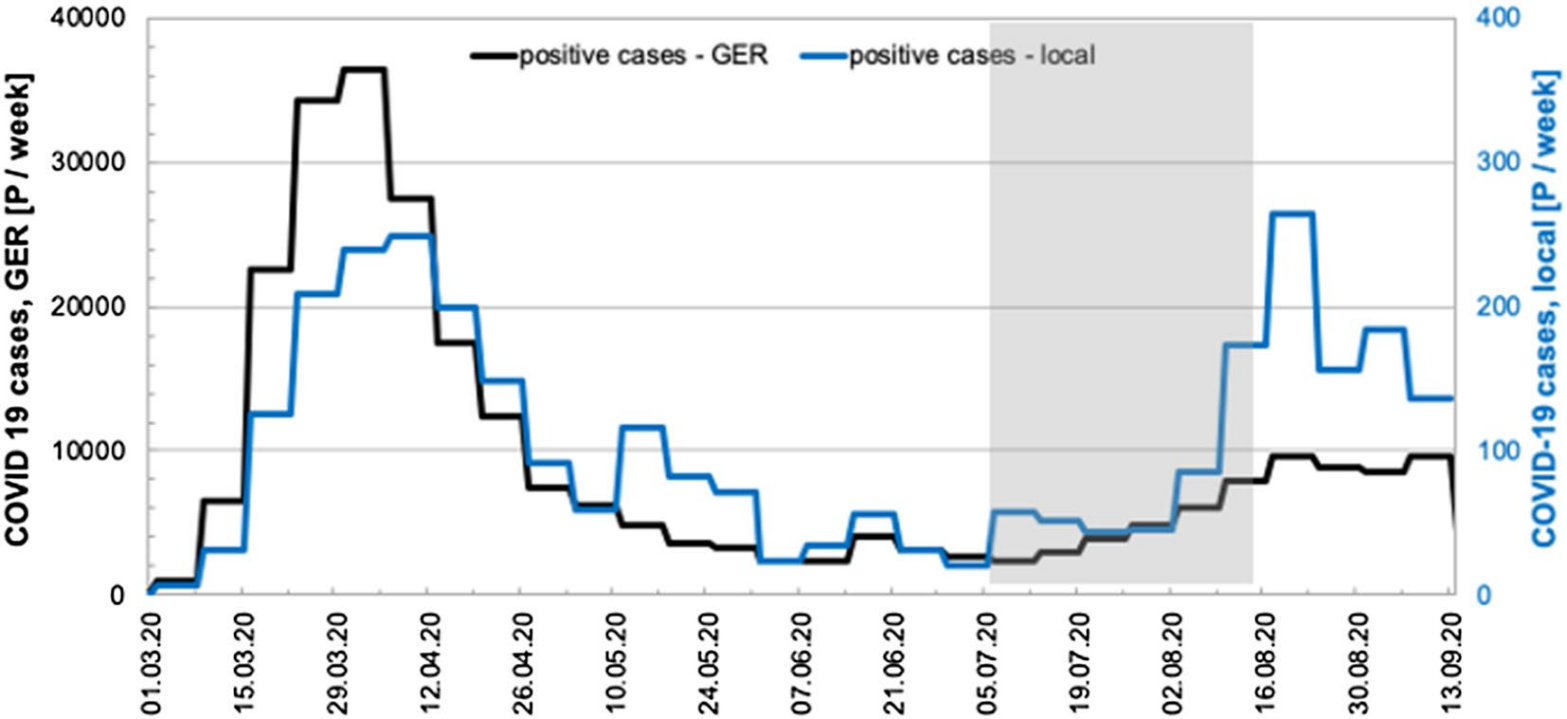
Graphical representation of the epidemiological data of COVID-19 cases in Germany (black line) and the city of Frankfurt am Main (blue line). The grey area indicates the time of the summer school vacation in the State of Hesse.

We investigated the largest metropolitan region in southern Germany (Frankfurt am Main) using three different sampling points in the catchment of the two large WWTPs (Table 1) and this data was compared to the COVID-19 cases in the area. Epidemiological data on COVID-19 in the studied area were retrieved from the publicly available repository of the Robert Koch Institute (https://survstat.rki.de/Default.aspx). Figure 1 shows the positive COVID-19 cases in Germany compared to the positive cases of the city of Frankfurt am Main, as reported weekly.

**Table 1:** Overview of the studied WWTPs in the city of Frankfurt am Main with the 85% percentile influent volume per day for the period 15.06–31.08.2020 and population equivalents (PE).

Location	PE	85% percentile influent volume (m^3^/d)
Frankfurt am main, niederrad/griesheim	1350.000	121.875/152.030
Frankfurt am main, sindlingen	470.000	64.085

The black line indicates the numbers in Germany with a high peak in March 2020 and the subsequent decline after the contact restrictions were imposed on the 22^nd^ of March. The blue line represents the COVID-19 cases of the city of Frankfurt am Main with 150 to 200 cases per week during the first peak in March and an increase of COVID-19 cases end of July. Frankfurt did not have a severe outbreak in the spring, with reported incidences not higher than 36 per 100.000 people for the first week of April. The grey bar symbolizes the summer vacation of all schools in Hesse, starting on the 06^th^ of July and lasting until the 14th of August. It marks a period with increased travelling within Germany and Europe, which resulted in similar or even slightly higher numbers of COVID-19 cases in the middle of August than in April. In contrast, the overall trend for Germany only saw a slight increase in cases during that time. Additionally, it is necessary to mention that during July and August, the German government started free-of-cost testing for people returning from other countries and risk regions to control returnees from holiday. This may have also led to an increase in the reported positive COVID-19 case in the summer months.

### Comparison of the results from April and August

**Figure 2:**
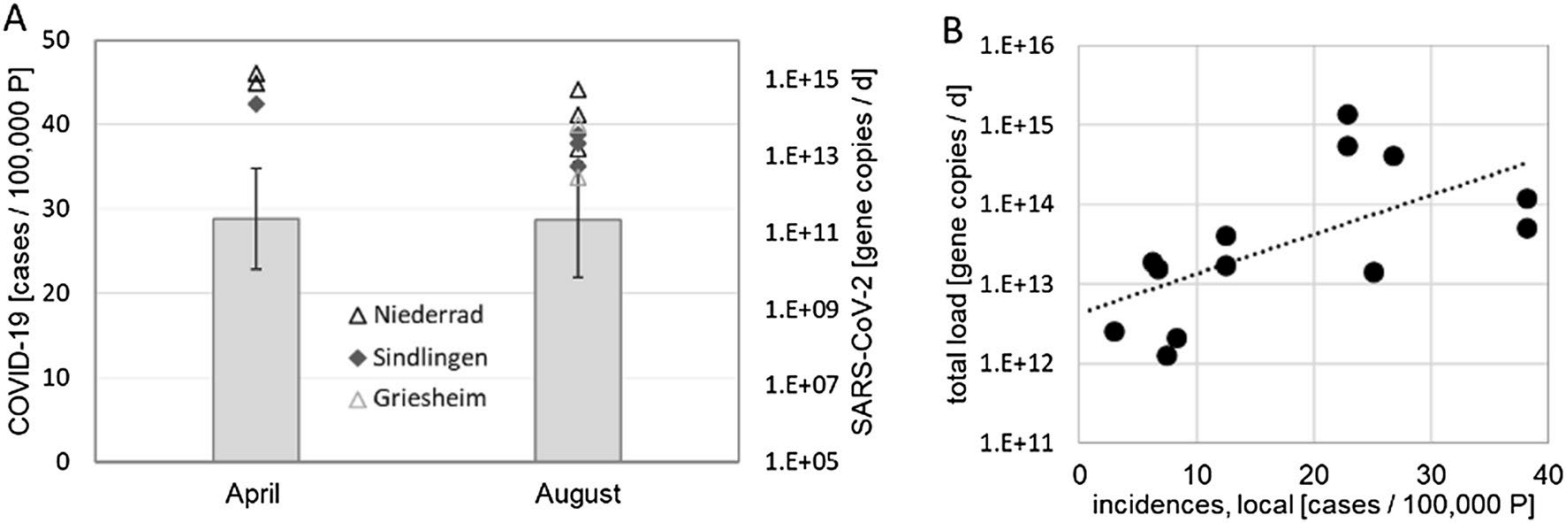
(**A**) Comparison of the results from April and August 2020, bars represent a three week average around the maximum number of cases per 100.000 persons (P), symbols refer to the respective SARS-CoV-2 RNA loads; (**B**) Correlation between the total load of SARS-CoV-2 gene copies (sum of the loads to both WWTPs) and incidences in Frankfurt am Main (local).

The highest numbers of COVID-19 cases in the city of Frankfurt am Main occurred in April and August 2020, with 28 cases per 100.000 people as three-week average. In contrast, the trend throughout Germany showed a much higher first peak in April with 44 cases per 100.000 people at the maximum and only 10 in August. Therefore, we used the COVID-19 cases and the corresponding SARS-CoV-2 RNA loads to the WWTPs both in April and August to compare both time points of the pandemic (Fig. 2A).

Our results revealed that the load of SARS-CoV-2 RNA in the wastewater samples was only slightly higher in April with a maximum of 1.4 × 10^15^ copies/d compared to a maximum of 5.37 × 10^14^ copies/d in August for individual influent wastewater samples. Additionally, we evaluated the correlation between the incidences reported against the sum of the SARS-CoV-2 RNA load measured in all the samples (Fig. 2B). It is clearly visible that with the increase in incidences, a corresponding increase in the viral load was observed as also shown in the Spearman's rank correlation coefficient r_s_ of 0.7464 (p (2-tailed) = 0.00217). However, there is a certain scattering in the data, which might be due to the variance in the measured viral load for different sampling points, as previously reported^10,15^.

### SARS-CoV-2 RNA in sewage samples

The sum of the SARS-CoV-2 RNA mean loads based on the concentrations of the three different target genes (N-, S- and ORF1ab gene) in the sewage samples from the two WWTPs was compared to the reported positive tested COVID-19 cases in Frankfurt am Main (see Supplementary information, figure S.1). In the study period, we observed that the SARS-CoV-2 RNA loads ranged between 1.29 × 10^12^ copies/d on the 06.07.2020 as lowest and 1.63 × 10^15^ copies/d on the 21.04.2020 as highest. In June and July, the incidences were less than ten COVID-cases per 100.000 persons per week and the load ranged between 1.29 × 10^12^ and 1.91 × 10^13^ copies/d. The comparison with the COVID-19 cases revealed that the increase in the SARS-CoV-2 RNA load clearly preceded the reported cases with the first step-increase in the middle of July 2020.

### Multiple WWTPs in the same metropolitan area

**Figure 3:**
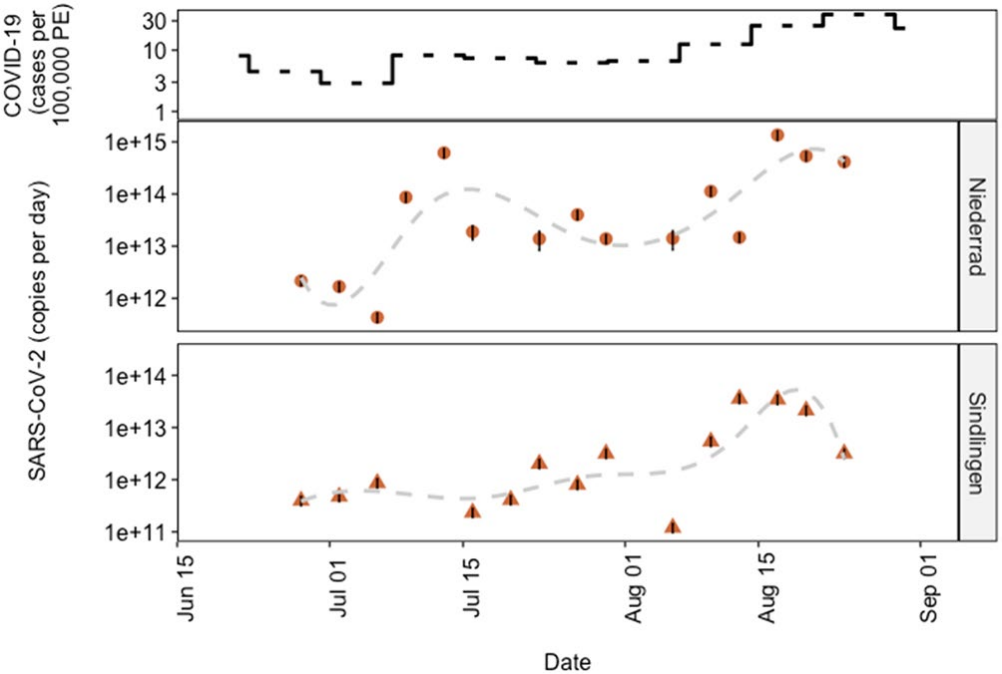
SARS-CoV-2 RNA loads analyzed with RT-qPCR in the influent of the two WWTP, i.e. Niederrad and Sindlingen, and the count of registered COVID-19 cases in the city of Frankfurt (top).

The Frankfurt metropolitan area has two main WWTPs, each receiving wastewater from different parts of the city and some neighbouring communities. We monitored the influent of both the WWTPs, to determine whether there is a significant difference in the viral load of the sewage or in its dynamic. After the first peak period of reported positive cases during March and April (Fig. 1), the daily load of SARS-CoV-2 RNA to the WWTP Niederrad indicated a slow increase over time, with two distinct peaks on the 13^th^ of July (i.e. 6.16 × 10^14^ copies/day) and the 17^th^ of August (i.e. 1.36 × 10^15^ copies/day) (Fig. 3). The increase in load in August is in line with the increase of COVID-19 cases in the studied area. However, the daily load of SARS-CoV-2 RNA in the influent of the WWTP Sindlingen was lower than the one in the influent of the WWTP Niederrad, remaining very similar until the middle of August with values between 1.20 × 10^11^ copies/day and 3.17 × 10^12^ copies/day. The values increased by a factor of 10 in August to a maximum value of 3.59 × 10^13^ copies/day when the COVID-19 cases also increased (Fig. 3). The results of the measurements in the influent to the WWTP Sindlingen also revealed a drop in virus load after the peak value of 3.59 × 10^13^ copies/day on the 13^th^ of August 2020. We detected a certain SARS-CoV-2 virus load in all the studied samples during these two months for both WWTP influents, with the highest load in the middle of August. The highest detected load also coincided with the end of the summer vacation season in the State of Hesse.

Additionally, to the reported loads, the concentrations of SARS-CoV-2 RNA in the influents to the WWTPs are provided in the Supplementary Information in Figures S.3, and S.4. The concentration of SARS-CoV-2 RNA ranged from 2.0 × 10^3^ copies/L to 3.0 × 10^6^ copies/L in the influent of the WWTP Niederrad and 3.0 × 10^3^ copies/L to 3.0 × 10^5^ copies/L in the influent of the WWTP Sindlingen. The concentrations showed very similar trends in comparison to the loads. Even though, load-based data is always preferred when dealing with wastewater, for SARS-CoV-2 the measured concentrations could already provide good approximations of the relative trends given that enough data points are available and no comparison with other samples is required.

**Figure 4:**
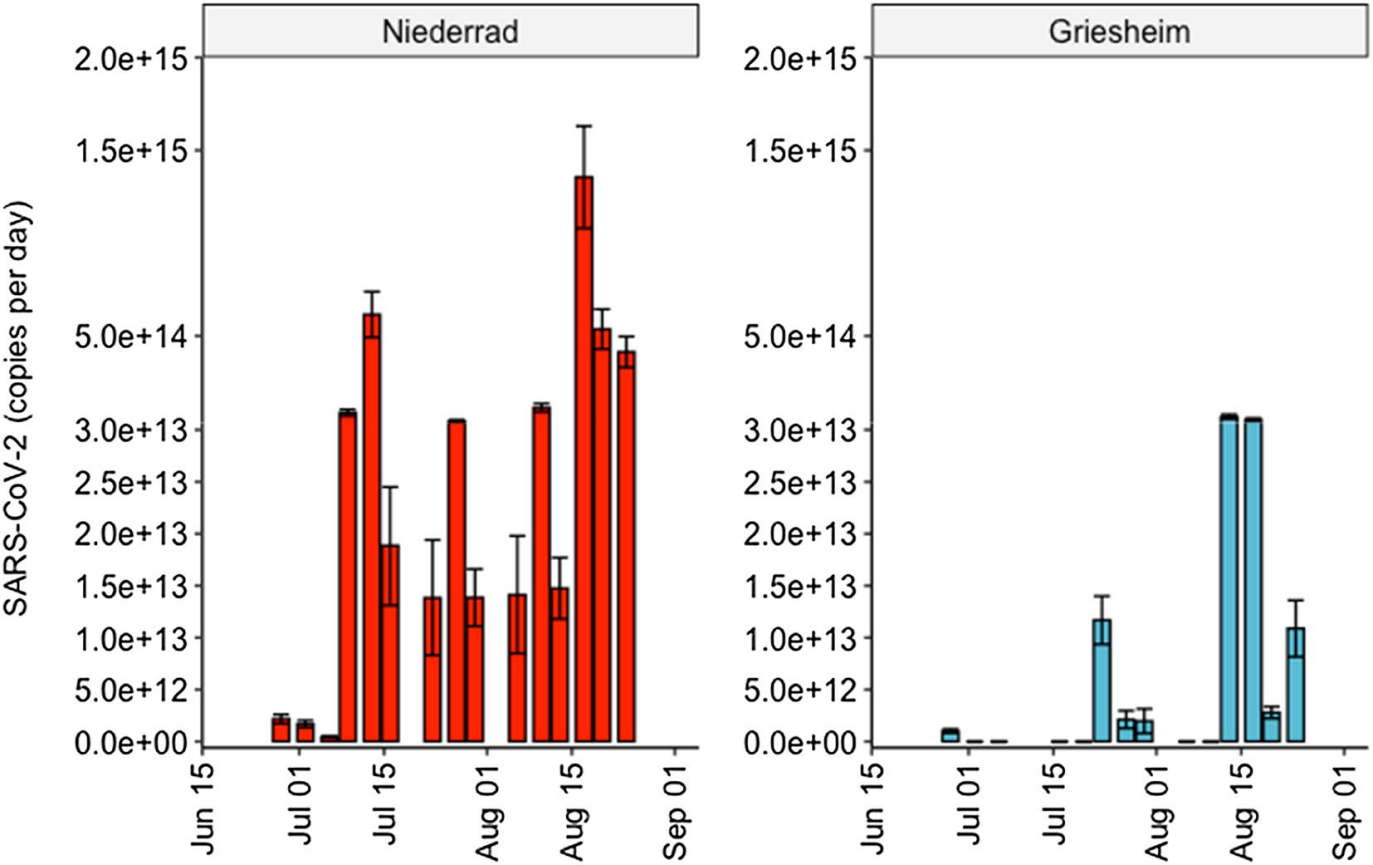
Load of SARS-CoV-2 target genes observed at the sampling point Griesheim and the WWTP Niederrad, respective concentrations of these two sampling points are provided in the Supplementary Information in Figures S.3 and S.5.

The WWTP Niederrad also receives sewage from Griesheim (see Supplementary information, figure S.5), a part of the Frankfurt metropolitan area, through a separate canal where sampling was possible. Therefore, to generate more localized information, we separately monitored sewage from Griesheim as well. Interestingly, we did not detect any SARS-CoV-2 RNA in a few samples from Griesheim during the study period. From the 14 sampling days, six samples showed results of SARS-CoV-2 RNA below the limit of detection (LOD), especially at the beginning of the study in June and July 2020, which corresponds well with the low number of COVID-19 cases reported during this time. Like other sampling points, a moderate increase of SARS-CoV-2 RNA load in the wastewater from Griesheim was observed, with a specific peak in August when the load was as high as 6.48 × 10^13^ copies/day (Fig. 4).

## Discussion

Our results exhibit the epidemiological patterns in the Frankfurt metropolitan area, where WBE indicated an increase of COVID-19 cases through an increase in the virus load to the WWTPs two weeks earlier than the case reports from the health sector. This result underlines that when cases resurged in August, our WBE analysis could have foreshadowed the increase in positive cases by 10 to 14 days. Similar observations have been reported in other studies^10,13,14^, showing temporal alignment between the COVID-19 cases and the measured concentrations of the SARS-CoV-2 RNA in wastewater. Previous studies of WBE SARS-CoV-2 surveillance in Europe focused on a random sampling of different WWTPs. However, similar long-term studies of WBE of SARS-CoV-2 in Europe indicated similar SARS-CoV-2 RNA concentrations in the wastewater^10,13^. For Germany, there is only one known study that conducted SARS-CoV-2 measurements in wastewater^15^. In this study, the authors measured the SARS-CoV-2 RNA concentration in nine WWTPs in western Germany and reported loads between 1 × 10^11^ to 1 × 10^13^ SARS-CoV-2 copies/day for these WWTPs. However, this study reports only on samples from one single time-point. Our data maps the trend of the SARS-CoV-2 concentration in the raw wastewater, also allowing the comparison with the dynamic of infections. Additionally, the comparison of COVID-19 cases and SARS-CoV-2 load (Fig. 2) suggests that the sometimes even controversially discussed number of cases going unreported in the first wave of the pandemic was not significantly higher in April than in August for the Frankfurt area, even though the number of tests differed significantly.

It is also important to note that previous studies vary not only in the sample volume, but also in the protocol of concentration of the virus and as well as the chosen target genes for analysis, which also comprises the use of different primer sets. For this reason, a comparison of SARS-CoV-2 virus loads and concentrations from other WWTPs with the ones reported in this study is not recommended, and preferably, the focus should lie on evaluating the trend of the virus load over time as the more significant and striking information.

Due to the rapid development of the pandemic and the need for testing, several companies have designed qRT-PCR assays to detect SARS-CoV-2. However, the performance of these assays may vary, esp. since they are designed for medical diagnostics and not for the use with environmental samples such as wastewaters^16,17^.

The results for the different target genes (i.e. ORF1ab, S and N gene) used in this study underlines (see Supplementary Information, figure S.2) the necessity to analyze more than one target gene for better confidence and reliable results. We observed variations in the amplification of the different target genes (see Supplementary Information, figure S.2) for different samples, suggesting the plausible presence of PCR inhibitors and a difference in sensitivity of the target genes to the PCR inhibitors. It is important to note that nucleic extracts from wastewater samples are generally known to contain both organic and inorganic inhibitors that could also affect viral recovery efficiency and subsequently, virus detection. Other studies have also reported variation in the results due to selected target genes and primers^10,12,15^.

Previously, studies have reported that wastewater with a higher density of particles generally yielded lower virus loads, especially with the focus on enveloped viruses due to greater adsorption capabilities to the solid fraction in the wastewater^18,19^. Therefore, it is important to estimate the viral recovery efficiency, as also suggested in other studies^17,20^. In our work, wastewater samples were spiked with MS2 phages as an internal control. The recovery efficiency varied between 12 to 89% without significant differences between the recovery efficiency across the different sampling points. We cannot exclude that PCR inhibitors had been present, which could also have affected the recovery efficiency of MS2 phage. Lu et al.^21^ investigated the impact of various methods for concentration of the viral RNA on viral RNA recovery efficiency. In general, Enterobacteria phages T3 and MS2 demonstrated higher mean recoveries compared to murine hepatitis viruses, spiked porcine epidemic diarrhea viruses and mengoviruses^21^. The recovery rates reported in the literature vary between 0–21.4%^22^ as lowest for SARS-CoV-1 and 85.5 ± 24.5 for Enterobacteria phages MS2^19^.

Our study showed that WBE with the detection of SARS-CoV-2 is a promising method for monitoring local developments. However, there is still the need for more long-term studies to understand and improve the sensitive steps of the analytical methods and gain more knowledge about the reliability of the trends in wastewater samples. The investigated samples in this study included almost exclusively dry weather samples, as the summer month July and August 2020 in Germany were hot and dry without heavy rain events. In future studies, it is thus also necessary to further consider the impact of sewer retention times, stormwater runoff events and the influence of wash-out on SARS-CoV-2 virus loads.

## Material and methods

### Sewage samples

24 h time-proportional composite samples were collected twice a week between April 2020 and August 2020 at three different sampling points from two wastewater treatment plants (WWTP), located in Frankfurt am Main, Germany. The WWTP Niederrad/Griesheim is designed for a population equivalent (PE) of 1.350.000, the WWTP Sindlingen for 470.000 PE. These two WWTPs receive the sewage of approximately 1.200.000 people, the remaining PE can be allocated to commercial and industrial discharges. Table 1 also includes the 85% percentiles of the influent volumes. 24 h composite samples (i.e. from 7 to 7 am) were collected with automatic flow proportional samplers installed at the three sampling points. Three sampling points were: (1) influent of the WWTP Niederrad, (2) influent of the WWTP Sindlingen, and (3) the sewage sample for Griesheim, a part of the Frankfurt metropolitan area, from a separate sewer before it merged with the influent of the WWTP Niederrad. During sampling, the temperature in the autosamplers was kept at 4 °C and the following day early morning (around 8 am) samples were transported at 4 °C to the lab. Immediately on arrival, (approx. around 9 am) samples were processed. Overall, we processed 44 samples, including 17 samples from the WWTP Niederrad sampling point, 14 from the WWTP Sindlingen sampling point, and 13 samples from the sampling point in Griesheim.

For each sampling location, one litre of the untreated wastewater was spiked with 200 µL MS2 phage (Thermo Fisher Scientific) as an internal control and filtered through a 0.45 µm electronegative membrane filter. The filters were divided and stored at minus 80 °C prior to further downstream analysis.

RNA was extracted from the filter samples using the Fast RNA Blue Kit (MP Biomedicals) according to the manufacturer’s protocol and eluted with 100 µL of RNase free buffer. This RNA was used as a template for RT-qPCR. The concentration was measured using a Qubit 3.0 Fluorometer (Thermo Fisher Scientific).

### qPCR analysis

The RNA was analyzed using the TaqPath COVID-19 RT-PCR Kit (Thermo Fisher Scientific) with a QuantStudio 3 Thermal Cycler. This kit includes primer pairs targeting the N-, S- and ORF1ab genes were used as a multiplex assay. Details about the kit are provided in the Supplementary Information. Each qPCR run was performed in triplicates with 50 µL volume, with 12.5 µL TaqPath 1-Step Multiplex Master Mix (4X), 2.5 µL COVID-19 Real Time PCR Assay Multiplex and 25 µL nuclease free water. To the reaction mix, 10 µL of purified and extracted viral RNA were added. Thermal profiles are provided in the Supplementary Information (Table 1). Reactions were considered positive if the cycle threshold was below 40 cycles, otherwise negative (i.e. no detection of the SARS-CoV-2 RNA in the sample). The limit of detection was 10 copies per RT-qPCR reaction. In each qPCR run, multiple SARS-CoV-2 RNA positive controls, a MS2 phage control (to determine the RNA recovery efficiency and as internal control) of different known concentrations and a negative control were included, further details are provided in the Supplementary Information. The RT-qPCR abundance data were analyzed in R, using ggplot2^23^ (v0.9.3.1), further details about the PCR efficiencies, threshold and baseline setting are provided in the Supplementary Information. For calculating the daily load of SARS-COV-2 RNA in the influent of the WWTP, daily readings of the respective flow volumes provided by the WWTP operator were used. The obtained RT-qPCR data in SARS-CoV-2 RNA copies per microliter were transformed to SARS-CoV-2 RNA copies per liter of sample and multiplied by these daily influent wastewater flow volumes (see also Table 1 for the 85% percentiles of the influent flow volumes for the period June—August 2020).

## Supplementary Information

Supplementary Information

## References

[1] WHO. Naming the Coronavirus Disease (COVID-19) and the Virus That Causes It. 2020. URL https://www.who.int/emergencies/diseases/novel-coronavirus-2019/technical-guidance/naming-the-coronavirus-disease-(covid-2019)-and-the-virus-that-causes-it (2020).

[2] Kataki S, Chatterjee S, Vairale MG, Sharma S, Dwivedi SK (2021). Concerns and strategies for wastewater treatment during COVID-19 pandemic to stop plausible transmission. Resour. Conserv. Recycl..

[3] Chen Y, Chen L, Deng Q, Zhang G, Wu K, Ni L, Yang Y, Liu B, Wang W, Wei C, Yang J, Ye G, Cheng Z (2020). The presence of SARS-CoV-2 RNA in the feces of COVID-19 patients. J. Med. Virol..

[4] Ren J, Li D, Wang C, Wu J, Wang Y, Sun Y, Zhang Q, Wang Y, Chang X (2020). Positive RT-PCR in Urine from an asymptomatic patient with novel coronavirus 2019 infection: a case report. Infect. Dis..

[5] Foladori, P., Cutrupi, F., Segata, N., Manara, S., Pinto, F., Malpei, F., Bruni, L., & La Rosa, G. SARS-CoV-2 from faeces to wastewater treatment: What do we know? A review. *Sci. Total Environ.* **2020**, 140444 (2020). 10.1016/j.scitotenv.2020.140444 PMC7311891 32649988

[6] Hovi T, Shulman LM, Van Der Avoort H, Deshpande J, Roivainen M, De Gourville EM (2012). Role of environmental poliovirus surveillance in global polio eradication and beyond. Epidemiol. Infect..

[7] Hellmér M, Paxéus N, Magnius L, Enache L, Arnholm B, Johansson A, Bergström T, Norder H (2014). Detection of pathogenic viruses in sewage provided early warnings of hepatitis a virus and norovirus outbreaks. Appl. Environ. Microbiol..

[8] Daughton CG (2020). Wastewater surveillance for population-wide covid-19: the present and future. Sci. Total Environ..

[9] Hata A, Honda R (2020). Potential sensitivity of wastewater monitoring for SARS-CoV-2: comparison with norovirus cases. Environ. Sci. Technol..

[10] Medema G, Heijnen L, Elsinga G, Italiaander R, Brouwer A (2020). Presence of SARS-coronavirus-2 RNA in sewage and correlation with reported COVID-19 prevalence in the early stage of the epidemic in The Netherlands. Environ. Sci. Technol. Lett..

[11] Sherchan SP, Shahin S, Ward LM, Tandukar S, Aw TG, Schmitz B, Ahmed W, Kitajima M (2020). First detection of SARS-CoV-2 RNA in Wastewater in North America: a study in Louisiana, USA. Sci. Total Environ..

[12] Ahmed W, Angel N, Edson J, Bibby K, Bivins A, O’Brien JW, Choi PM, Kitajima M, Simpson SL, Li J, Tscharke B, Verhagen R, Smith WJM, Zaugg J, Dierens L, Hugenholtz P, Thomas KV, Mueller JF (2020). First confirmed detection of SARS-CoV-2 in untreated wastewater in Australia: a proof of concept for the wastewater surveillance of COVID-19 in the community. Sci. Total Environ..

[13] La Rosa G, Iaconelli M, Mancini P, Bonanno Ferraro G, Veneri C, Bonadonna L, Lucentini L, Suffredini E (2020). First detection of SARS-CoV-2 in untreated wastewaters in Italy. Sci. Total Environ..

[14] Nemudryi, A., Nemudraia, A., Surya, K., Wiegand, T., Buyukyoruk, M., Wilkinson, R., & Wiedenheft, B. *Temporal Detection and Phylogenetic Assessment of SARS-CoV-2 in Municipal Wastewater*; preprint; Epidemiology. org/10.1101/2020.04.15.20066746 (2020). 10.1016/j.xcrm.2020.100098 PMC7457911 32904687

[15] Westhaus, S., Weber, F.-A., Schiwy, S., Linnemann, V., Brinkmann, M., Widera, M., Greve, C., Janke, A., Hollert, H., & Wintgens, T. Detection of SARS-CoV-2 in raw and treated wastewater in Germany–suitability for COVID-19 surveillance and potential transmission risks. *Sci. Total Environ.* **2020**, 141750 (2021). 10.1016/j.scitotenv.2020.141750 PMC7434407 32861187

[16] Farkas K, Hillary LS, Malham SK, McDonald JE, Jones DL (2020). Wastewater and public health: the potential of wastewater surveillance for monitoring COVID-19. Curr. Opin. Environ. Sci. Health.

[17] Kitajima M, Ahmed W, Bibby K, Carducci A, Gerba CP, Hamilton KA, Haramoto E, Rose JB (2020). SARS-CoV-2 in wastewater: state of the knowledge and research needs. Sci. Total Environ..

[18] Nordgren J, Matussek A, Mattsson A, Svensson L, Lindgren P-E (2009). Prevalence of norovirus and factors influencing virus concentrations during one year in a full-scale wastewater treatment plant. Water Res..

[19] Ye Y, Ellenberg RM, Graham KE, Wigginton KR (2016). Survivability, partitioning, and recovery of enveloped viruses in untreated municipal wastewater. Environ. Sci. Technol..

[20] Thompson JR, Nancharaiah YV, Gu X, Lee WL, Rajal VB, Haines MB, Girones R, Ng LC, Alm EJ, Wuertz S (2020). Making waves: wastewater surveillance of SARS-CoV-2 for population-based health management. Water Res..

[21] Lu D, Huang Z, Luo J, Zhang X, Sha S (2020). Primary concentration—the critical step in implementing the wastewater based epidemiology for the COVID-19 pandemic: a mini-review. Sci. Total Environ..

[22] Wang X-W, Li J-S, Guo T-K, Zhen B, Kong Q-X, Yi B, Li Z, Song N, Jin M, Xiao W-J (2005). Concentration and detection of SARS coronavirus in sewage from Xiao Tang Shan Hospital and the 309th Hospital. J. Virol. Methods.

[23] Wickham, H. *ggplot2: Elegant Graphics for Data Analysis* (Springer International Publishing: Imprint: Springer, 2016). 10.1007/978-3-319-24277-4.

