# Supplementary material for: Long-term monitoring of SARS-CoV-2 RNA in wastewater of the Frankfurt metropolitan area in Southern Germany
Source: Sci Rep. 2021 Mar 8;11:5372. doi: 10.1038/s41598-021-84914-2 (PMC7940401; doi:10.1038/s41598-021-84914-2)
Supplement: Supplementary file 1 — Supplementary Information [file 41598_2021_84914_MOESM1_ESM.docx]

**Supplementary Information**

**Long-term monitoring of SARS-CoV-2 RNA in wastewater of the Frankfurt metropolitan area in Southern Germany**

Shelesh Agrawal^1,*^, Laura Orschler^1^, Susanne Lackner^1^

*^1^ Technical University of Darmstadt, Institute IWAR, Chair of Wastewater Engineering, Franziska-Braun-Straße 7,64287 Darmstadt, Germany*

*** *E-mail: s.agrawal@iwar.tu-darmstadt.de, Phone: +49 615 116 20309, Fax: +49 615 116 20305.*

**Methods**

**qPCR analysis**

We performed qPCR analysis using the TaqPath COVID-19 RT-PCR Kit (Thermo Fisher Scientific)^1^ which includes: (1) TaqPath COVID‐19 Assay Multiplex, which contains three primer/probe sets specific to different SARS-CoV-2 genomic regions (i.e. N gene, S gene and Orf1ab gene) and primers/probes for bacteriophage MS2. (2) MS2 Phage Control – RNA control, having a concentration of 10^6^ copies per µl, to verify the efficacy of the RNA extraction and the absence of inhibitors in the PCR reaction. (3) TaqPath COVID‐19 Control – Positive SARS-CoV-2 RNA control that contains targets specific to the SARS-CoV-2 genomic regions targeted by the assays. The manufacturer (Thermo Fisher Scientific) has not publicly released the primers/probe sets sequences, therefore, we do not have access to the information related the primers/probe sets sequences and the length of the PCR products.

Table 1:Information about dyes corresponding to each target gene.

| **Gene** | **Dye** | **Quencher** |
| --- | --- | --- |
| ORF1ab | FAM | QSY |
| N Protein | VIC | QSY |
| S Protein | ABY | QSY |
| MS2 (Internal Control) | JUN | QSY |

In case of the positive control, we included triplicates of the four different concentration (i.e. 1x10^1^, 2x10^1^, 2x10^2^, 2x10^3^ copies per reaction) of the TaqPath COVID‐19 positive control for each qPCR run. For MS2 phage internal control triplicates of the three different concentration (i.e. 2x10^2^, 2x10^3^, 2x10^4^ copies per reaction) were also included in each qPCR run. For the SARS-CoV-2 positive control and MS2 phage internal control, each reaction contained 12.5 µL TaqPath 1-Step Multiplex Master Mix (4X), 2.5 µL COVID-19 Real Time PCR Assay Multiplex, 33 µL nuclease free water, and 2 µL of positive or internal control. Triplicates of negative controls were also included in each run, each reaction contained 12.5 µL TaqPath 1-Step Multiplex Master Mix (4X), 2.5 µL COVID-19 Real Time PCR Assay Multiplex, and 35 µL nuclease free water.

Ct values of positive control dilutions were plotted against known concentrations of the SARS-CoV-2 positive control and MS2 phage internal control, to generate standard curves. The start baseline value was set at 5 and threshold cycle (Ct) values were determined manually while adjusting the threshold to be above any background signal and within the exponential phase of the fluorescence curves. Primer efficiencies were

95.32 ± 9.09% for N, 91.09 ± 13.84% for S, 86.75 ± 1.8% for Orf1ab, and 95.92 ± 17.16% for MS2 phage (n = 8 runs, mean ± sd). The slopes of the standard curves for the quantification were −3.43 ± 0.26 for N, −3.55 ± 0.46 for S, −3.68 ± 0.28 for Orf1ab, and −3.42 ± 0.25 for MS2 phage. Respective Y-intercept values were 38.17 ± 0.83, 37.04 ± 0.39, 38.55 ± 0.86, and 37.89 ±0.77. The SARS-CoV-2 loads detected in the samples are presented without correcting for recovery efficiencies.

Table 2: PCR protocol

| **Step** | | **Temperature** | **Duration** |
| --- | --- | --- | --- |
| Hold-Stage | | 25 °C | 2 min |
| Hold-Stage | | 53 °C | 10 min |
|  |  | 95 °C | 2 min |
| PCR-Stage | 45 cycles | 95 °C | 15 s |
|  |  | 60 °C | 1 min |

**Results**

**Recovery efficiency of the MS2 phages results**

The recovery efficiency of the concentration and extraction procedure performed in triplicates, was determined by using the non-enveloped *Enterobacteria* MS2 phage. It showed an average recovery in the range of 11.53 - 89.11 %, with a median value of 42.40%.

Table 3: Recovery efficiency of the MS2 phages for each sample for each sampling point.

| **Sampling point** | **Samples** | **Recovery Efficiency** |
| --- | --- | --- |
| Influent of the WWTP Niederrad | Sample 1 | 27.66 % |
|  | Sample 2 | 38.57 % |
|  | Sample 3 | 11.53 % |
|  | Sample 4 | 42.40 % |
|  | Sample 5 | 50.32 % |
|  | Sample 6 | 34.65 % |
|  | Sample 7 | 32.40 % |
|  | Sample 8 | 51.91 % |
|  | Sample 9 | 67.02 % |
|  | Sample 10 | 89.11 % |
|  | Sample 11 | 42.40 % |
|  | Sample 12 | 41.20 % |
|  | Sample 13 | 48.39 % |
|  | Sample 14 | 13.64 % |
|  | Sample 15 | 19.04 % |
|  | Sample 16 | 14.75 % |
|  | Sample 17 | 42.01 % |
| Influent of the WWTP Sindlingen | Sample 1 | 15.32 % |
|  | Sample 2 | 54.33 % |
|  | Sample 3 | 33.73 % |
|  | Sample 4 | 63.04 % |
|  | Sample 5 | 61.98 % |
|  | Sample 6 | 42.03 % |
|  | Sample 7 | 37.19 % |
|  | Sample 8 | 65.04 % |
|  | Sample 9 | 43.07 % |
|  | Sample 10 | 43.81 % |
|  | Sample 11 | 21.91 % |
|  | Sample 12 | 71.54 % |
|  | Sample 13 | 31.96 % |
|  | Sample 14 | 43.14 % |
| Sewage sample for Griesheim | Sample 1 | 19.90 % |
|  | Sample 2 | 64.03 % |
|  | Sample 3 | 21.01 % |
|  | Sample 4 | 43.54 % |
|  | Sample 5 | 42.77 % |
|  | Sample 6 | 24.13 % |
|  | Sample 7 | 11.98 % |
|  | Sample 8 | 54.92 % |
|  | Sample 9 | 44.53 % |
|  | Sample 10 | 53.73 % |
|  | Sample 11 | 63.64 % |
|  | Sample 12 | 25.98 % |
|  | Sample 13 | 44.72 % |

**Overall SARS-CoV-2 RNA load in the influent wastewater and positive tested COVID-19 cases in the city of Frankfurt am Main**


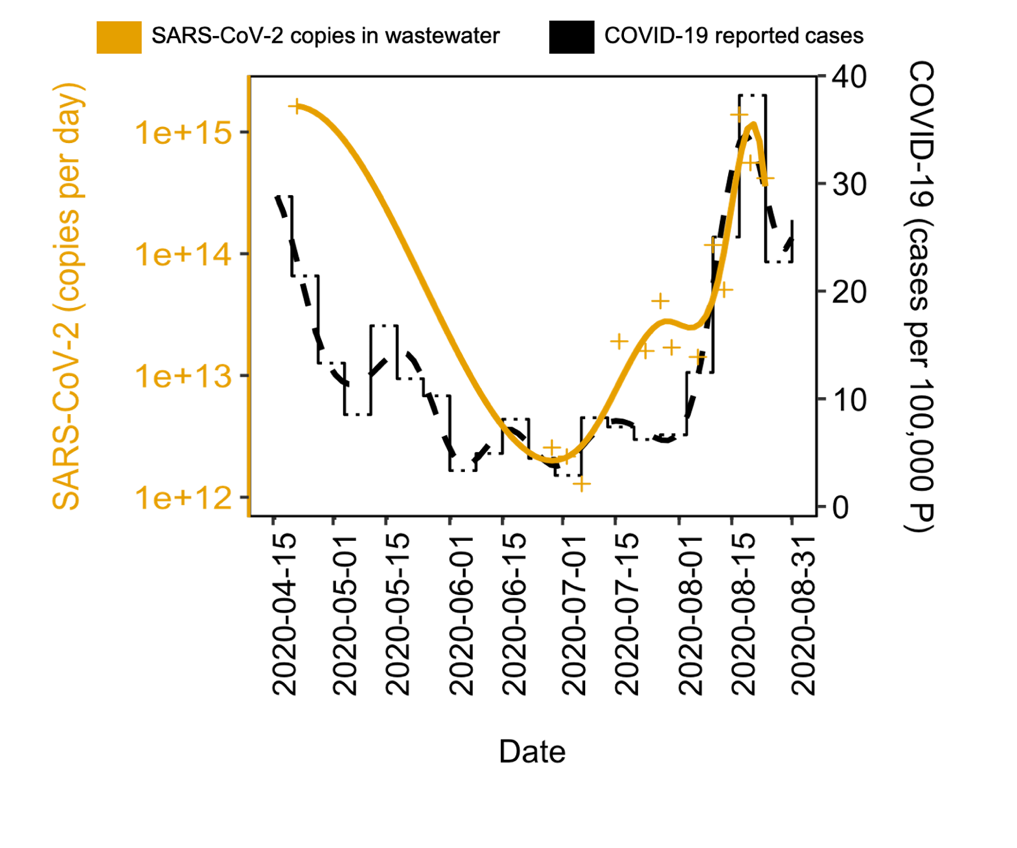


S.figure 1: SARS-CoV-2 load as sum of the two WWTP influents as analyzed with RT-qPCR in comparison to the positive tested COVID-19 cases in the city of Frankfurt am Main.

**Impact of chosen target genes**

S.figure 2 shows that different target genes performed differently, especially until the middle of July, when less COVID-19 positive cases were reported. Moreover, variation in the performance of target genes differed with different samples. For example, in initial Niederrad samples, we detected SARS-CoV-2 ORF1ab gene copies only. Whereas, for Sindlingen samples ORF1ab and S gene copies were detected. Based on the results, we recommend targeting multiple genes for SARS-CoV-2 monitoring in wastewater.


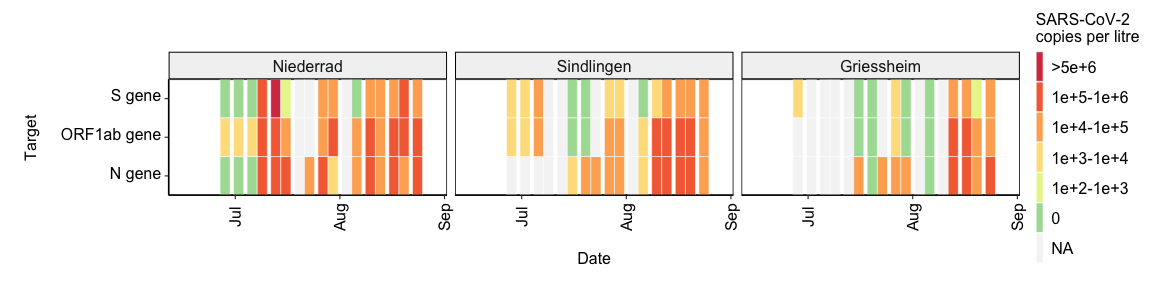


S.figure 2: Heatmap showing the SARS-CoV-2 concentration measured for each sample from each sampling point based on three different target genes (S gene, ORF1ab gene, and N gene).NA: Not detected.

**Concentrations of SARS-CoV-2 RNA in the untreated wastewaters**

S.figure 3: Concentrations of SARS-CoV-2 RNA in the influent of the WWTP Niederrad as determined by real-time qPCR

S.figure 4: Concentration of SARS-CoV-2 RNA in influent of the WWTP Sindlingen as determined by real-time qPCR

S.figure 5: Concentration of SARS-CoV-2 RNA in the wastewater at sampling point Griesheim as determined by real-time qPCR

References

(1) *TaqPath COVID‑19 CE‑IVD RT‑PCR Kit Instruction for Use*; MAN0019215; Thermofisher Scientific.
